# Xanthophylls from the Sea: Algae as Source of Bioactive Carotenoids

**DOI:** 10.3390/md19040188

**Published:** 2021-03-27

**Authors:** Antia G. Pereira, Paz Otero, Javier Echave, Anxo Carreira-Casais, Franklin Chamorro, Nicolas Collazo, Amira Jaboui, Catarina Lourenço-Lopes, Jesus Simal-Gandara, Miguel A. Prieto

**Affiliations:** 1 Nutrition and Bromatology Group, Analytical and Food Chemistry Department, Faculty of Food Science and Technology, Ourense Campus, University of Vigo, E-32004 Ourense, Spain; antia.gonzalez.pereira@uvigo.es (A.G.P.); paz.otero@uvigo.es (P.O.); javier.echave@uvigo.es (J.E.); anxocc@uvigo.es (A.C.-C.); chamorro1984@gmail.com (F.C.); nicolascollazojimenez@gmail.com (N.C.); jarb.amira@gmail.com (A.J.); lopes@uvigo.es (C.L.-L.); 2 Centro de Investigação de Montanha (CIMO), Instituto Politécnico de Bragança, Campus de Santa Apolonia, 5300-253 Bragança, Portugal

**Keywords:** carotenoids, xanthophylls, natural compounds, algae, bioactive, health

## Abstract

Algae are considered pigment-producing organisms. The function of these compounds in algae is to carry out photosynthesis. They have a great variety of pigments, which can be classified into three large groups: chlorophylls, carotenoids, and phycobilins. Within the carotenoids are xanthophylls. Xanthophylls (fucoxanthin, astaxanthin, lutein, zeaxanthin, and β-cryptoxanthin) are a type of carotenoids with anti-tumor and anti-inflammatory activities, due to their chemical structure rich in double bonds that provides them with antioxidant properties. In this context, xanthophylls can protect other molecules from oxidative stress by turning off singlet oxygen damage through various mechanisms. Based on clinical studies, this review shows the available information concerning the bioactivity and biological effects of the main xanthophylls present in algae. In addition, the algae with the highest production rate of the different compounds of interest were studied. It was observed that fucoxanthin is obtained mainly from the brown seaweeds *Laminaria japonica*, *Undaria pinnatifida*, *Hizikia fusiformis*, *Sargassum* spp., and *Fucus* spp. The main sources of astaxanthin are the microalgae *Haematococcus pluvialis*, *Chlorella zofingiensis,* and *Chlorococcum* sp. Lutein and zeaxanthin are mainly found in algal species such as *Scenedesmus spp.*, *Chlorella spp.*, *Rhodophyta* spp., or *Spirulina* spp. However, the extraction and purification processes of xanthophylls from algae need to be standardized to facilitate their commercialization. Finally, we assessed factors that determine the bioavailability and bioaccesibility of these molecules. We also suggested techniques that increase xanthophyll’s bioavailability.

## 1. Introduction

In recent years, consumer demand for naturally sourced products to promote health and reduce disease has grown steadily [1]. This demand has entailed an increased interest in new natural sources of food, pharmaceutical, and cosmetic products [2,3]. In this context, the marine environment has been considered a potential reservoir of natural compounds [4]. Among the organisms present in this environment, it is worth highlighting algae. Algae constitute a polyphyletic group of photosynthetic primary producers organisms, which represent an interesting source of chemical components with high-value biological activities. [5]. Although the total number of algal species is unknown, it is thought to vary between one and ten million [6].

The high value of algal extracts is due to their large number of molecules such as carbohydrates, proteins, peptides, lipids (including oils and polyunsaturated fatty acids, PUFAs), minerals, iodine, phenols (polyphenols, tocopherols), alkaloids, terpenes, and pigments (as chlorophylls, carotenoids, and phycobilins) [7,8]. Within these compounds, one of the groups with greater interest are pigments due to the concentrations in which they are present, these being higher than that of other compounds such as phenolic compounds. In fact, algae are considered pigment-producing organisms. They have a great variety of pigments, which can be classified into three large groups: chlorophylls, carotenoids, and phycobilins. Therefore, different carotenoids (CA) profiles can be used as a medium for algal classification [9]. In this way, a first classification of the algae allows us to make a division according to the size of the algae (microalgae or macroalgae) and the following divisions according to their tones, among other characteristics. As a result, the first group comprises greenish algae (Cyanophyceae), green algae (Chlorophyceae), diatoms (Bacillariophyceae), and golden algae (Chrysophyceae), among others. Meanwhile, the macroalgae family includes red (Rhodophyta), brown (Ochrophyta), and green algae (Chlorophyta) [10,11,12]. This diversity of species and, therefore, of its chemical compositions is interesting, since once compounds are properly isolated or extracted from algae, they may show a diverse range of biological activities, such as antioxidant, antimicrobial, anticancer, anti-allergic, antiviral, and anticoagulant activities, among others [7,8]. This diversity of biological activities implies that there is also a significant variety of potential applications in human health, agriculture, and in food and cosmetic industries [4], in which its application depends on its chemical composition.

On an industrial scale, the most interesting species are those that produce high percentages of CA. CA are usually located in chloroplasts or stored in vesicles and a cytoplasmic matrix of plants, algae, photosynthetic bacteria, and some fungi [9]. All CA are tetraterpenes, which are compounds that have a skeleton composed of 40 carbon atoms conjugated in polyene chains [9]. They are classified into two main groups: (i) compounds that have a hydrocarbon long chain known as carotenes and (ii) compounds that have an oxygen atom in its structure, known as xanthophylls. The first group includes α-carotene, β-carotene, lycopene, and phytoene, among others. The most representative molecules of the second group are fucoxanthin, astaxanthin, lutein, zeaxanthin, and β-cryptoxanthin. This difference in its structure makes xanthophylls more polar than carotenes due to the presence of oxygen in the form of methoxy, hydroxy, keto, carboxy, and epoxy positions. However, except for lutein, they are still non-polar compounds [13]. Its structure with alternating double bonds is responsible for many of its biological functions, being the main function in photosynthetic organisms to act as accessory pigments for the capture of light in photosynthesis, and to protect photosynthetic machinery against self-oxidation [14]. However, despite the wide diversity of molecules in the carotenoid family, with more than 700 compounds currently known, only about 30 CA have a significant role in photosynthesis [13]. In recent years, numerous studies have highlighted CA multiple effects on human health due to their antioxidant properties, preventing the damage caused by oxidative stress and therefore declining the risk of chronic diseases [14,15]. However, the biological properties of CA are not limited to their antioxidant properties. The scientific literature has shown CA actions as anti-tumor [16,17,18], anti-inflammatory [19,20], neuroprotective, antimicrobial, antidiabetic, and antiobesity [21,22]. Therefore, algae have several CA with market interest (β-carotene, fucoxanthin, astaxanthin, lutein, zeaxanthin, and violaxanthin), representing a natural and sustainable source of these compounds [9].

Among the xanthophylls of interest is fucoxanthin, which is one of the most abundant marine CA, accounting for approximately 10% of the total production of natural CA [23]. It is found in abundant concentrations in the chloroplasts of several brown seaweeds, such as *Laminaria japonica*, *Undaria pinnatifida*, *Sargassum fusiformis*, in several species belonging to the genera *Sargassum* (*Sargassum horneri*) and *Fucus* (*Fucus serratus, Fucus vesiculosus*) and in diatoms (*Bacillariophyta*) [9,24,25,26]. Another xanthophyll of interest is astaxanthin (AS), which is a red pigment. AS is considered a potent antioxidant as it has about ten times more antioxidant activity than other CA [27]. The main natural sources of this pigment are the microalgae *Haematococcus pluvialis*, *Chlorella zofingiensis,* and *Chlorococcum* sp. [28]. *H. pluvialis* is a single-celled green freshwater alga. It is the richest source of natural AS and is already produced on an industrial scale [26]. Procedures have been technologically advanced to grow *Haematococcus* containing 1.5–3.0% AS dry weight [27,29]. The richest source of β-carotene is the halotolerant green microalgae *Dunaliella salina*, accumulating up to 10% of it based on the dry weight of the microalgae [30,31]. When *H. pluvialis* and *D. salina* are cultivated in extreme conditions (such as high salinity, high luminosity, or lack of nutrients), AS and β-carotene, respectively, can reach more than 90% of the total carotenoids [7]. Lutein and zeaxanthin are pigments found in algal species such as *Scenedesmus spp.*, *Chlorella spp.*, *Rhodophyta* spp., or *Spirulina* spp. respectively [32]. Esteban et al., 2009 [33], reported that red algae (*Rhodophyta*) show a common carotenoid pattern of β-carotene and one to three xanthophylls: lutein, zeaxanthin, or anteraxanthin. *Corallina elongata* and *Jania rubenseran* were the only algae that contained anteraxanthin as the main xanthophyll. *Spirulina platensis* (strain pacifica) microalgae is a source of β-cryptoxantine, β-carotene, and zeaxanthin. β-cryptoxantine is a pigment that can also be found in plants [34]. The siphonaxanthin content in green algae such as *Umbraulva japonica, Caulerpa lentillifera,* and *Codium fragile* constitutes about 0.03%–0.1% of the dry weight [35]. The cyanobacteria *Synechococcus* sp. strain PCC7002 produces a monocyclic myxoxanthophyll, which is identified as Myxol-2 Fucoside (Myxoxanthophyll), in addition to producing other CA such as β-carotene, zeaxanthin, and sinecoxanthin [36]. The CA composition in cyanobacteria is very different from that of other algae, including for example β-carotene, zeaxanthin, myxol pentosides, and echineone [32].

**Figure 1:**
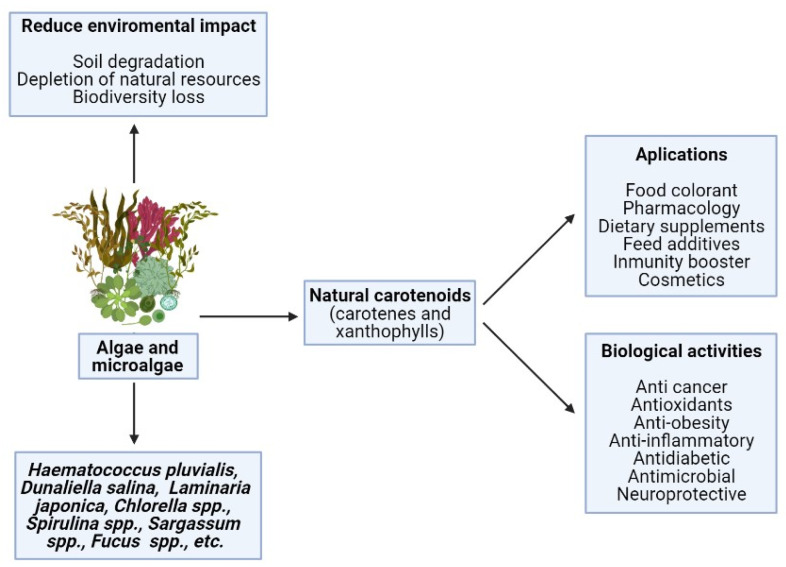
Positive effects on human health and industrial applications of carotenoids from natural sources.

Animals should get all these CA through the diet, as they are unable to synthesize them. CA are commonly incorporated as dietary supplements, feed additives, and food colorants in several sorts of food, such as dairy products and beverages, and also in the pharmaceutical and cosmetic industries [37]. As shown in Figure 1, CA have a high repertoire of commercial applications due to their multiple biological properties. Among the most notable applications are cosmetic, nutraceuticals, pharmaceutical purposes, and other human applications.

Attributable to the various positive activities on human health and the multiple industrial applications of CA, global demand continues to increase. It is estimated that in 2026, the CA market will grow to USD 2.0 billion, registering an annual growth rate for CA of 4.2% [38]. The most relevant and important pigments on the market today are β-carotene and AS, followed by lutein, lycopene, and canthaxanthin [13,31]. So far, most commercial CA are artificially produced. However, the strong global interest in food of natural origin that is safe, healthy, and environmentally friendly has increased the demand for natural sources of CA [22]. Algae and algal extracts are a sustainable option for CA and have numerous benefits in comparation to alternative natural sources. For instance, its cultivation and production is cheap, easy, and ecological, its removal has higher yields and is simple, and raw materials are not scarce, nor are there seasonal limitations [32,39,40]. In order to obtain high concentrations of a certain compound, culture conditions and environmental stress can be modified to manipulate the biochemical composition of microalgae [39]. However, under optimal growth conditions, the concentration of CA pigments is often too low to produce microalgal-based pigments, making it economically unviable [13,40]. To improve its economic viability, it is vital to explore and understand how environmental factors and the integration of nutrients into the environment affect the production of compounds. Understanding how the metabolic pathways of species vary according to the culture conditions, the co-production and accumulation of multiple compounds in microalgae will be improved [41]. The purpose of this review is to highlight the impact of xanthophylls from algae on human health, and to study the factors affecting the feasibility of their production and use as a sustainable alternative source of CA in the coming years.

## 2. Main Xanthophylls Present in Algae

From examining the findings, algae are a raw material of interest due to their pigment content and the potential bioactivities they possess. However, at present, relatively few species are used for such purposes since their exploitation at an industrial level is scarce. Table 1 lists some cases on algae exploitation to obtain high value xanthophylls. It includes information about the main algae species producing xanthophylls and their applications together with the main extraction techniques used to obtain the high-value molecules. The amount obtained in each case provides necessary information to estimate whether the process is viable.

### 2.1. Fucoxanthin

**Figure 2:**
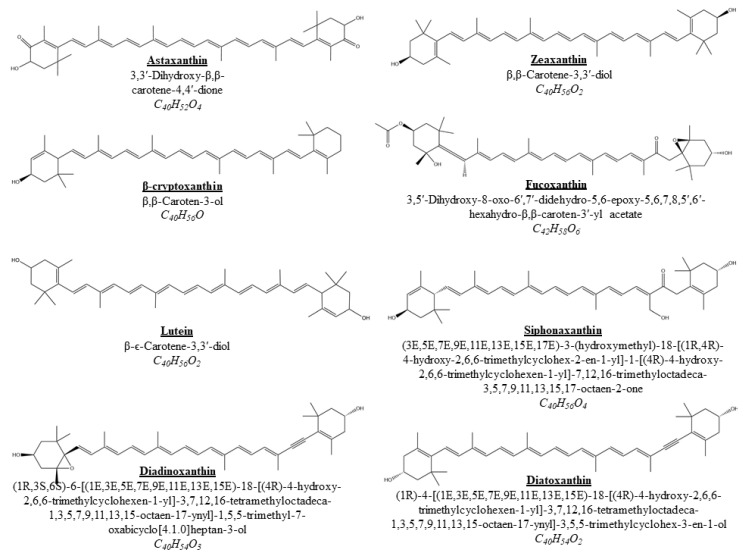
Chemical structure of the main xanthophylls present in algae [82].

Fucoxanthin (FU) (Figure 2) is produced by many algae as a secondary metabolite. It is present in the chloroplasts of eukaryotic algae and is involved in the process of photosynthesis performed by algae, which is thought to be more efficient than the photosynthesis of plants [77]. This molecule is considered one of the most abundant pigments in brown algae, and it represents up to 10% of the total CA found in nature [78]. It has been studied primarily in microalgae and brown macroalgae from several families such as *Undaria*, *Laminaria, Sargassum*, *Eisenia*, *Himathalia*, *Alaria,* or *Cystoseira* [79,80]. FU has a chemical structure derived from carotene but with an oxygenated backbone. In addition, this compound has several different functional groups such as hydroxyl, carboxyl, epoxy, and carbonyl moieties, and it also has an allenic bond [25]. FU is orange to brown in color, and it is responsible for the coloration of algae from the Phaeophyceae family. This lipophilic pigment absorbs light in a range from 450 to 540 nm, which translates in the blue-green to yellow-green part of the visible spectrum, and it behaves as the primary light-harvesting CA for many algae transferring energy to the chlorophyll–protein complexes with high efficiency thanks to its unique CA structure [81].

Many bioactivities have been reposted regarding FU. Several articles have been published about its antioxidant, anticancer, anti-inflammatory, antimicrobial, antihypertensive, anti-obesity, antidiabetic, and anti-angiogenic activities, and also its photoprotective and neuroprotective effects (Table 1) [79,83,84,85,86,87,88,89,90,91]. Considering all these properties, FU has a great potential for applications in all sectors, from supplements and enriched foods to anti-aging cosmetics and to the pharmaceutical sector in the development of new innovative drugs for all kinds of pathologies including different types of cancer. For all these reasons, the FU market is expected to keep growing and reach 120 million dollars by 2022 [92].

Even though the artificial laboratory synthesis of FU is possible, it is a very expensive process that makes the extraction of FU from algae so appealing. However, the extraction and purification processes of FU from algae need to be standardized to facilitate its future commercialization and incorporation to new profitable products on the market [48]. Nevertheless, some companies already overcame these problems, and valuable products with FU have reached the market. For example, food supplements with FU intended to contribute to the loss of weight and improve eye, brain, liver, and joint health, are being sold with the commercial name of ThinOgen^®^ and Fucovital^®^. These products can be found in the form of oils or microencapsulated powders [93]. Furthermore, FU is being studied to help combat cancer-related diseases, showing different anticancer mechanisms of action, such as inhibition of cell proliferation, induction of apoptosis, cell cycle arrest, an increase of intracellular reactive oxygen species, and anti-angiogenic effects [84,88,94,95,96,97]. Many studies have been made applying FU extracts to human cell lines, such as human bronchopulmonary carcinoma cell line NSCLC-N6, erythromyeloblastoid leukemia cell line K562, and the human lymphoblastoid cell line TK6, all with positive results [98]. Similar results were observed in prostate cancer (PC-3) cells, leukemia cells (HL-60), and cervical adenocarcinoma cells (HeLa). In addition, in vivo studies were also performed. For example, in mice, the administration of FU suppressed tumor growth of primary effusion lymphoma, sarcomas, and osteosarcoma [51,55,96]. Due to its anti-inflammatory activity, FU is also being tested to prevent and treat inflammatory-related diseases, thanks to fucoxanthin’s strong antioxidant capacity and gut microbiota regulation [99], and its capacity to inhibit the production of nitric oxide, which is one of the determinants of inflammation in cells [100]. Some examples of FU incorporations in several food matrixes can already be found in the literature such as fortified yogurt [52] and milk [53], enriched canola oil [101], baked products such as scones [46], and even ground chicken breast meat [102].

### 2.2. Astaxanthin

Astaxanthin (AS) is a ketocarotenoid that fits in the group of terpenes and is formed from five carbon precursors, isopentenyl diphosphate, and dimethylallyl diphosphate. It is produced by a restricted number of algae (mainly microalgae), plants, bacteria, and fungi [103]. In microalgae, this compound is a secondary CA, which means that its accumulation in cytosolic lipid bodies ensues exclusively beneath environmental stress or adverse culture conditions, such as high light, high salinity, and nutrient deprivation. Despite this, algae represent the most important natural source of this compound in the aquatic food chain [104].

The commercial manufacture of this pigment has conventionally been executed by chemical synthesis. However, current studies proved that some microalgae might be the most capable source for its industrial biological production [105]. The best known and most used microalgae for its production are *Haematococcus pluvialis* and *Chlorella zofingiensis* [106]. *Haematococcus pluvialis* is one of the organisms with the highest concentrations of AS; thus, it is the main industrial source for the natural production of this compound [107]. It is common to reach yields of 38–40 g/kg (3.8–4%) of dried algae, and its scale at an industrial level is possible due to the high reproduction rate of this microalga [78,79]. The amount of AS found in cells corresponds to 85–95% of the total CA content; thus, it is relatively easy to purify it from the remaining CA [108]. Other species such as *C. zofingiensis* have also been studied, but the content of AS found was 50% AS of total CA, being the other main CA canthaxanthin and adonixanthin [109]. The extraction of AS, which is a lipophilic compound, can be carried out with organic solvents and oils, and it is common to combine its extraction with solvents with other types of extractions such as enzymatic or microwave extraction [107].

This compound is known as one of the most potent antioxidants; its capacity is due to the large amount of conjugated double bonds (thirteen). Different studies confirm that its antioxidant capacity is 65 times more potent than that produced by ascorbic acid; 10 times stronger than β-carotene, canthaxanthin, lutein, and zeaxanthin; and 100 times more effective than α-tocopherol, all of which are antioxidants used routinely [108]. For this reason, various products containing AS are already available on the market in various forms including oils, tablets, capsules, syrups, soft, creams, biomass, or ground [107]. An example is AstaPure^®^ (Algatech LTD) produced from the microalgae *H. pluvialis.* Moreover, the consumption as a supplement does not represent any risk of toxicity, since the human body is not capable of transforming AS into vitamin A [107]. In 2019, the European Food Safety Authority (EFSA) has established an acceptable daily intake of 0.2 mg per kg body weight [110]. However, in order to be used as a food additive, more studies are still required due to stability, conservation, handling, and storage problems in this type of matrix [111].

AS has also anti-inflammatory activity, which is mainly due to its antioxidant properties and has been concerned in meliorate lifestyle-related illnesses and dealing health. AS additionally has anti-aging activity [105]. These beneficial effects have been demonstrated for both animals and humans [107].

### 2.3. Lutein

Chemically, lutein (LU) is a polyisoprenoid with 40 carbon atoms and cyclic structures at each end of its conjugated chain. Therefore, it has a similar structure to zeaxanthin (explaining below), differing from it in the site of the double bond in one ring, giving three chiral centers compared to the two of zeaxanthin [112]. LU is already used regularly in sectors such as cosmetics, pharmaceuticals, and food, which is mainly due to its color and bioactivities, and its anticancer properties are worth noting [61]. In fact, different studies demonstrate the antitumor effects of LU. For example, it was found that oral LU supplementation reduced the influence of ultraviolet irradiation by diminishing acute inflammatory responses and hyperproliferative rebound induced by ultraviolet rays [113]. In addition, this compound is widely known for its preventative effects against age-related macular degeneration and cataracts [62]. These health-promoting properties of LU along with its potential as a natural food colorant have led to improved research on the potential of LU as a high-value nutraceutical ingredient [114].

In general terms and for healthy people, food is a proper source of LU, and it does not require being added in a balanced diet, as it is safe to consume 60 mg/day for an adult of 60 kg [115]. This dietary contribution of LU is mainly due to the consumption of vegetables. However, algae is being considered as a new reservoir of lutein [59]. Among them, the best source at the commercial level is microalgae, especially those belonging to the *Chlorella* genus. This alga is an effective source of LU production, and it is safer than that of chemical origin whose use remains questionable. For this reason, the growth optimization studies of this alga are gaining interest owing to the high growth rates of the alga, along with their high pigment content. Several studies analyze the effect of LU production under different microalgae growth conditions in bioreactors. In most of them, the optimized parameters are the concentration of nitrate, ammonium, and urea in the batch [60,61]. However, the cultivation conditions of other newer species such as *Scenedesmus almeriensis* have also been optimized to increase their LU production. In this case, the contribution of nutrients has a lesser effect due to the high tolerance of this microalga to varied ranges of temperature, pH, salinity, and nutrient concentration [63]. Other widely studied species for its high content of LU are *D. salina* and *Galdieria sulphuraria* [59]. Mostly, it is still necessary to reduce costs regarding the growth and extraction process of LU from microalgae to be profitable. For this, it is not only necessary to optimize the consumption of nutrients, but also to analyze the subsequent processes such as harvesting and drying that entail large energy costs. In this regard, the currently available studies seem to indicate that the best option may be tubular photobioreactors [114,116].

### 2.4. Zeaxanthin

Zeaxanthin (ZEA) is a structural isomer of LU. Both isomers are usually found in various foods, being mainly present in green leafy vegetables and algae [117]. It is formed by a polyene chain with 11 conjugated double bonds and ionone rings. The ionone rings have a hydroxyl group that can attach to the fatty acids during esterification [118]. This compound, as well as some derivatives (meso-zeaxanthin), has a high antioxidant effect due to its chemical structure and distribution of the bonds. Furthermore, it also has a powerful anti-inflammatory effect attributable to the down-regulated expression of several inflammatory mediator genes. Consequently, these compounds may also be used in cancer prevention, as tumors are considered inflammatory diseases. Therefore, their use in chemotherapy may be of great interest [119]. Other bioactivities include photoprotection as well as the prevention and treatment of some eye diseases such as progress of macular degeneration and cataracts [120,121]. Moreover, ZEA has been proved to possess anti-tyrosinase activity, an enzyme associated with the production of melanin. Therefore, the inhibition effect of ZEA on this enzyme may avoid the formation of skin spots, which point to the use of this pigment as a whitening agent [122]. Hence, ZEA is a CA with promising nutraceutical implications.

Humans are not able to synthetize ZEA, as there is no biosynthetic pathway for this compound; thus, it has to be obtained from the diet. For this reason, its extraction from natural sources including vegetables, plants, macroalgae, cyanobacteria, and microalgae is of great interest [123]. There are several species of microalgae that produce this pigment. One of them is *Dunaliella salina*, which has also been genetically modified to increase its yield under all growth conditions, reaching 6 mg ZEA per gram of algae [124]. Other species that synthesize ZEA include *Spirulina, Corallina officinalis, Cyanophora paradoxa* and *Glaucocystis nostochinearum* [117]. These organisms can accumulate ZEA in a concentration up to nine times higher than traditional sources of this compound such as red peppers. This is the case of *Chlorella ellipsoidea*. In addition, algae have the advantage over plant matrices that the ZEA present in algae is in free form, while in plants, it is present as mono and diesters of ZEA [66]. As a consequence, numerous studies show the development of protocols to obtain ZEA from microalgae on a large scale [125]. Moreover, the production of this compound can be increased by varying the conditions in which algae cultivation takes place. One option is to increase photosynthetic irradiance over that required for the saturation of photosynthesis [117].

### 2.5. Minor Carotenoids

In addition to FU, AS, LU, and ZEA, algae can synthetize low amounts of other CA that belong to the xanthophyll group. In this section, we assessed these minor molecules also susceptible to be exploited by the nutraceutical industry. These include β-cryptoxanthin, siphonaxanthin, saproxanthin, myxol, diatoxanthin, and diadinoxanthin. They are only present in some bacteria and marine algae.

#### 2.5.1. β-Cryptoxanthin

β-cryptoxanthin is an oxygenated CA with a chemical structure close to that of β-carotene, being the most important difference the higher polarity of β-cryptoxanthin. The interest of this compound shows a positive correlation between the intake of β-cryptoxanthin and the prevention of several diseases. In fact, this molecule is characterized by having provitamin A activity, anti-obesity effects, antioxidant activities, and anti-inflammatory, and anti-tumor activity [126]. Furthermore, the influence of β-cryptoxanthin on some inflammatory markers is probably stronger than other CA [127]. This compound is much less common than β-carotene, and it can only be found in a small number of foods. Some of them are fruits and vegetables such as tangerines, red peppers, and pumpkin [128]. It is also possible to find this compound in algae, mainly in red algae due to its hue [68]. Its concentration on each product will depend on environmental factors such as season, processing techniques, and storage temperatures [126].

#### 2.5.2. Siphonaxanthin

Siphonaxanthin is a specific keto-carotenoid current in comestible green algae such as *Codium fragile, Caulerpa lentillifera*, and *Umbraulva japonica*, constituting around 0.1% of their dry weight [35]. This compound is present mainly in species belonged to the Siphonales order, which is characterized by grouping green algae inhabiting deep waters from both freshwater and marine environments [67].

Some studies have been carried out with this molecule, showing the potential beneficial effects on health, including anticancer activities and its suitability in the treatment of leukemia, with even better results than those obtained with FU [35]. This greater capacity to produce an apoptosis-inducing effect may be due to the fact that siphonaxanthin, unlike FU, does not have an epoxide or an allenic bond in its structure, but it does contain an additional hydroxyl group at carbon 19 that might be responsible for this activity [129]. Other activities include anti-angiogenic, antioxidant and anti-inflammatory. The anti-inflammatory effect is due to the suppression of mast cell degranulation in vivo as it alters the functions of lipid rafts by localizing in the cell membrane and inhibiting the translocation of immunoglobulin E (IgE) / IgE receptor (FcεRI) to lipid rafts [130].

#### 2.5.3. Saproxanthin

Saproxanthin is an uncommon and recently described natural CA found in algae, bacteria, and archaea [131], being bacteria the main source. Chemically, it is a tetraterpene with a CA β-cycle additionally hydroxylated at C3 as one end group and simple hydration of the most distant double bond at the other termination of the compound [132]. Therefore, this compound is also a xanthophyll. It was initially reported and described by Aasen and Jensen in *Saprospira grandis* [67]. This compound is a potent antioxidant. It is produced by algae with the aim to protect itself from the activated oxygen produced by light [133]. In vitro studies have shown its pure form pose high antioxidant activity against lipid peroxidation in the rat brain homogenate model and a neuroprotective effect of l-glutamate toxicity [133,134].

#### 2.5.4. Myxol

Myxol is a derivative of γ-carotene and is present in different forms in nature (free or combined with fucosides or nitrogen groups). Nevertheless, in the free state, it is found primarily in marine environments [67]. It should be noted that this pigment is glycosylated in the 2′-OH position instead of the usual position (1′-OH) of the molecule [36]. The main group of organisms that produce this compound are cyanobacteria [135]. Cyanobacteria were previously called myxophyceae, which is named after the characteristic molecule of this family [36]. Some cyanobacteria in which this pigment has been characterized are Anabaena and Nostoc [136]. Nonetheless, algae not only contain free myxol; thus, it is also possible to quantify some combined forms of myxol. One study detected the presence of pro-glyoxylate derivative compounds such as pro-2′-O-methyl-methylpentoside and 4-keto-myxol-2′-methylpentoside in freshwater algae *Oscillatoria limosa* [137]. All variants of this molecule have been proved to have antioxidant properties. In fact, its antioxidant activity is greater than that of other frequently used antioxidant molecules such as ZEA and β-carotene [138]. For example, one study was able to demonstrate significant antioxidant activities against lipid peroxidation in the rat brain homogenate model and a neuroprotective effect of l-glutamate toxicity [134]. Other in vitro studies have concluded that myxol might also be effective in strengthening biological membranes, reducing permeability to oxygen. Nonetheless, these novel and rare CA require meticulous assessments before their execution [138].

#### 2.5.5. Diatoxanthin

Diatoxanthin, a ZEA analogue, is a type of xanthophyll found in phytoplankton and diatoms. Diatoms are often called golden brown microalgae, due to their content of pigments, mainly CA, comprising FU, diadinoxanthin, and diatoxanthin [139]. These compounds have the function of serving as a protection system for algae against the harmful effects of light saturation. Thanks to its presence, the algae are able to quickly acclimatize to the difference in the amount of light received and therefore continue to carry out their vital functions without alterations [140]. Therefore, an effective way to increase the production of this compound, and hence its performance, is to increase the blue-light irradiation; 300 μmol photons m^−2^·s^−1^ is enough for *Euglena gracilis* [141].

#### 2.5.6. Diadinoxanthin

Similar to diatoxanthin, diadinoxanthin is present only in limited algal groups, including diatoms. In fact, these pigments might be considered as diatom-specific CA [73]. Both compounds are interrelated, since diadinoxanthin is the inactive precursor of diatoxanthin, and it can be transferred to the active compound very quickly when subjected to high light stress [140]. Diadinoxanthin, together with FU, can be obtained from neoxanthin. For this, it is necessary to have a simple isomerization of one of the allenic double bonds of neoxanthin molecule [74]. Its antioxidant activity is based on deepoxidized diadinoxanthin to diatoxanthin, which leads to reduction of the singlet oxygen inside the cell, avoiding cellular damage [142].

## 3. Mechanism of Action of Xanthophylls

### 3.1. Metabolism

The mechanism of action of xanthophylls is the specific binding through which the molecule produces its pharmacological effect. This effect will depend on the absorption, distribution, and metabolism of the compound, which are critical parameters of the pharmacokinetics of the xanthophylls. This can be seen in various studies that show the low presence of this type of compound in human tissues, which directly depends on their metabolism and intestinal absorption, and therefore, its bioavailability [143]. The metabolism of xanthophylls is poorly studied, especially for those that do not have provitamin A activity. Hence, more studies are needed to understand its metabolism and, therefore, be able to develop different applications according to the mechanism by which its biological activities occur.

In turn, this would allow the development of safe and effective applications in humans as well as increase its bioavailability [144]. For example, studies on FU metabolism revealed that this compounds itself is not present in plasma but rather its metabolites due to oxidative reactions that take place on FU in mammals. This reaction transforms both compounds into ketocarotenoids [145]. In addition, when FU is administered orally, it undergoes a process of hydrolysis at the intestinal level, giving rise to fucoxanthinol, while liver metabolization results in other active metabolites such as amarouciaxanthin A [146,147]. In fact, it was reported that dietary FU accumulated in the heart and liver as fucoxanthinol and in adipose tissue as amarouciaxanthin A, the latter being non-detectable by HPLC in human serum [148]. Therefore, the oral administration of this compound may only provide some bioactive metabolites, as it is completely metabolized. To release products that maintain its biological activities, it is necessary to develop alternatives that prolong its biological half-life [146], such as emulsions or encapsulations (Table 2).

A study carried out on rats reported that the pharmacokinetic parameters of AS only depend on the dose when it is administered intravenously due to the metabolism that takes place in the liver as a result of saturation of hepatic metabolism of AS [162]. As for AS metabolites described in humans, these are fundamentally 3-hydroxy-4-oxo-β-ionone and 3-hydroxy-4-oxo-7,8-dihydro-β-ionone [163]. The metabolization of AS after oral intake leads to 3-hydroxy-4-oxo-7,8-dihydro-β-ionol and 3-hydroxy-4-oxo-7,8-dihydro-β-ionone, being both compounds detected in plasma [164]. Several researchers hypothesize that the rate at which these reactions take place is determined by the structure of the ring, as well as by the length of the fatty acyl residue formed. Moreover, several enzymes, such as for example diacylglycerol acyltransferase 1, can catalyze the synthesis of AS esters in some strain. This is the case of the microalga *Haematococcus pluvialis* [165].

As for LU and its structural isomer, ZEA, studies carried out in humans have shown that both undergo an in vivo oxidation process that gives rise to several metabolites [166]. LU gives rise to a series of compounds (3′-epilutein, 3′-oxolutein) due to the presence of the enzyme that also mediated the conversion of fucoxanthinol to amarouciaxanthin A [167]. Other compounds such as 3-hydroxy-3′,4′-didehydro-β,γ-carotene and 3-hydroxy-2′,3′-didehydro-β,ε-carotene appear as result of acid hydrolysis in the stomach [168]. However, this compound is capable of remaining intact in its intact form in human ocular tissue due to the inability of the enzyme β-carotene-9′,10′-oxygenase to act on said organ. In this way, there is an extraordinary accumulation of these compounds in the ocular tissue, serving as a mechanism for the prevention of ocular diseases [169]. ZEA, being an isomer of LU, will undergo similar processes to LU. However, it is a much less studied molecule. In this way, ZEA will also be accumulated in the ocular tissue due to the inactivity of the enzymes responsible for the metabolism of ZEA in the organs of sight [170]. Therefore, to determine the bioavailability of LU it is necessary to quantify said metabolites, which also may have different bioactivities, with complementary studies.

### 3.2. Bioavailability and Bioaccessibility

Xanthophylls have been subjected to numerous studies due to its antioxidant activity and protective effect against several diseases [171]. In recent years, different studies have been carried out comparing the properties of synthetic CA with those of natural origin [172], noting that some of them can only be obtained from natural sources, where there is much more diversity. In addition, these CA obtained from algae can be co-extracted with other bioactive components such as polysaccharides or fatty acids. Therefore, the idea of incorporating CA in foods, nutraceuticals, or cosmetic products is of increasing interest due to their effective bioactive properties [173]. However, to develop and evaluate the viability of any food or cosmetic products that maintain these activities, it is necessary to know its bioactivity, bioavailability, and bioaccesibility [174]. These three parameters are influenced by several factors such as the food matrix; the type of cooking; the time of cooking; the CA involved; the presence of fats, fibers, proteins, and other nutrients in the diet; and the health or nutritional status in humans [175,176,177,178,179].

**Figure 3:**
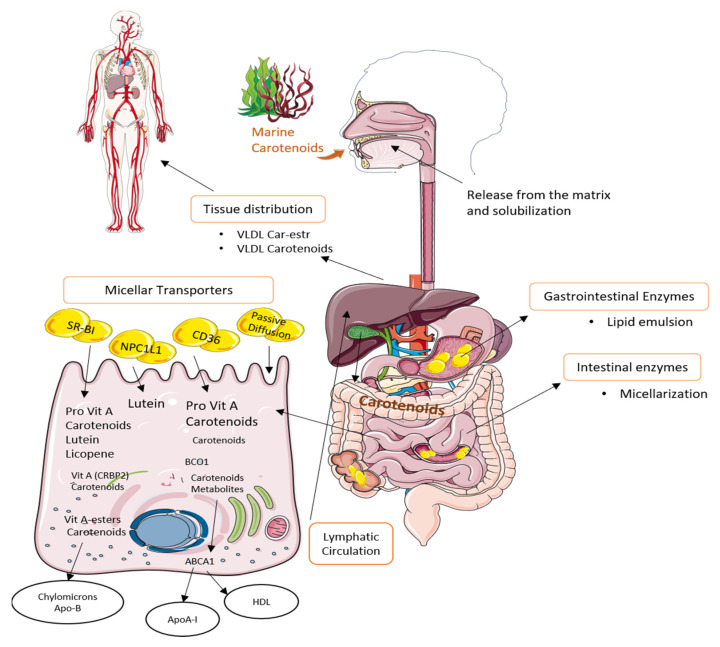
Uptake, transport, and secretion pathways of marine carotenoids in the human body.

In humans, once CA are ingested, they are released from the food matrix through the action of gastric enzymes and must be emulsified with lipids in order to improve their absorption [180]. Moreover, its absorption mechanism will be determined by the concentration in which the compound is present. At low concentrations, absorption is mainly due to the action of type 1 class B scavenger receptor, which also captures high-density lipoproteins, platelet glycoprotein 4, and NPC1-like intracellular cholesterol transporter 1 [181]. At high concentrations, the main mechanism is passive diffusion through mucosa [182]. Enzymes released in the duodenum will also play an important role in the absorption, since in this part of the small intestine, pancreatic lipase is released. This enzyme assists the formation of mixed micelles of emulsified droplets with CA. This process depends on the concentration of bile acids among others [183]. Once the micelles are formed, they pass into the blood. Then, micelles are taken up by enterocytes, in which metabolization takes places due to the presence of the enzyme β-carotene oxygenase. The non-metabolized CA, such as LU and ZEA, are incorporated into chylomicrons or low-density lipoproteins (LDL) and are transported to the liver where they can be eliminated by the bile or metabolized and secreted in very low-density lipoprotein (VLDL) or high-density lipoproteins (HDL) to the peripheral tissues, as it can be seen in Figure 3 [180,184].

All these absorption processes involve passing through membranes, which will be determined by the polarity of the membrane and the compounds. CA are frequently esterified with fatty acids, which decreases the polarity, so except for lutein, they are considered non-polar molecules. Among CA, xanthophylls have a bit higher polarity than carotenes. This is due to the small number of oxygen atoms in their structure (Figure 2). In addition, the polar groups of the molecules are at opposite ends of the molecule, so their forces cancel out. Therefore, the presence of hydroxyl groups makes them a bit more polar than carotenes, which do not contain oxygen but are still considered non-polar molecules [185]. CA polarity and flexibility seem to be correlate with bioaccessibility and uptake efficiency. This may be due to the fact that this type of CA presents a better affinity for lipid transporters and/or for plasma membranes, which would increase absorption [186]. Therefore, these compounds may be the CA with highest bioavailability. Different mechanisms have also been developed to increase the bioavailability of these compounds, of which the most common are the elaboration of emulsions or encapsulations.

#### 3.2.1. Fucoxanthin

Different in vitro, in vivo, and clinical studies show that FU digestion and absorption gives rise to metabolites such as fucoxanthinol. In a study carried out with mice, FU was transformed into fucoxanthinol in the gastrointestinal mucosa by deacetylation due to the action of lipase and cholesterol esterase enzymes. Then, the fucoxanthinol that reached the liver was transformed to amarouciaxanthin by deoxidation. As a result, fucoxanthinol could be detected in the heart, spleen, liver, and lung, and amarouciaxanthin could be found in adipose tissue [145,148]. During all this process, pH is a limiting factor since, as it was observed in an in vitro simulated digestion study, enzymes can be inactivated due to low pH and, consequently, FU would remain intact [187]. A study of the colonic fermentation of FU reported that 50% of FU can be metabolized by action of the human microbiota, ensuring that the compound is bioaccessible [187]. However, the absorption of FU is lower than the rest of the CA despite achieving better accumulation [188]. This may be due to digestion of the compound. In fact, FU supplementation in adults correlated with fucoxanthinol increase in serum [189]. A human trial carried out with FU extracted from *Undaria pinnatifida* concluded that after the supplementation of an extract with 6.1 mg of FU, FU could not be detected in blood, and the metabolite fucoxanthinol was at very low concentration, which confirms the limited intestinal absorption of FU [190]. In order to improve its absorption, different mechanisms have been developed, of which the most common encapsulation is in micelles or liposomes [149]. The best results are obtained when long or medium-chain triglycerides are used to carry out the encapsulation [152]. Encapsulation can also be done with chitosan-glycolipid nanogels, which increase FU bioavailability by 68% according to in vitro studies [153]. Other options include encapsulation with proteins such as zein and caseinate, which provide better stability to FU and enhance its antioxidant and anti-tumor activity compared to free FU [150]. Yet, human studies are scarce and contradictory, since numerous factors that influence bioavailability are reported, such as the dietary fiber of the food matrix; the interaction with other nutrients such as lipids and proteins; the solubility of the molecule; or the affinity with intestinal transporters.

#### 3.2.2. Astaxanthin

AS is considered the compound with the highest bioavailability among CA, followed by lutein, β-carotene, and lycopene [185]. However, its bioavailability depends on the type of matrix and on the stresses of this molecule in colonic Caco-2/TC7 cells [191]. A study carried out in an in vitro digestion model with human intestinal Caco-2 cells of three geometric isomers of AS conclude that the isomerization occurs at a gastrointestinal level, with the 13-cis-astaxanthin isomer showing the greater bioaccesibility and the higher concentrations in blood [192]. In human plasma, AS increases in a dose-dependent manner, achieving stimulation of the immune system, and decreasing oxidative stress and inflammation [193]. High doses (100 mg) present maximum levels of absorption at 11.5 h, while low doses (10 mg) reach them at 6.5 h [194]. Moreover, the bioavailability of said compound can be improved by emulsion with lipids, becoming between 1.7 and 3.7 times better compared to the reference formulation [195]. Other options include encapsulation with lipoprotein aggregates, maltodextrin, pectin, or chitosan [155]. Newer encapsulation methods have also been developed such as oleic acid–bovine serum albumin complexes nanoparticles [196], which are able to find products that, for example, use nanoemulsions with ascorbyl palmitate in sublingual application to favor the absorption and bioavailability of AS [158]. Nevertheless, as AS may be easily degraded by digestive acids, intake after digestion has shown increased levels of absorption [197]. Moreover, the consumption of AS in synergy with fish oil increased the lipid-lowering effects and increased phagocytic activity compared to the consumption of free AS [154]. On the contrary, sociological factors such as smoking habits also play an important role in bioavailability, since tobacco inhibits the bioavailability of AS [194]. AS has already been studied as dietary supplements in Europe, Japan, and the United States, demonstrating their safety in human clinical trials of up to 40 mg/day. Based on these data, the US Food and Drug Administration has approved AS from *H. pluvialis* for human consumption at 12 mg per day and up 24 mg per day for no more than 30 days [194].

#### 3.2.3. β-Cryptoxanthin

The bioaccesibility of various xanthophylls has been demonstrated in numerous studies. In this regard, an in vitro gastric simulation study proved that all-trans-β-cryptoxanthin has 31.87% of bioaccesibility that could be improved by modifying the nature of the matrix [198]. Additional studies suggest a mechanism for the digestion and intestinal absorption of β-cryptoxanthin in its free and esterified forms. The study was made in a digestion model with Caco-2 cells and intestinal cells clone Caco-2 TC7, reporting that β-cryptoxanthin is more bioaccessible than β-carotene, but having worse uptake with Caco-2 TC7 cells [199]. At present, this lack of knowledge makes this compound subject to controversy, since there are studies with disparate results. For example, some of the sources that were consulted state that serum β-cryptoxanthin bioavailability is greater than β-carotene measured in humans after dietary intake [200].

#### 3.2.4. Zeaxanthin

ZEA constitutes one of the three macular pigments, and it is characterized by having a preventive effect in age-related eye diseases [201]; consequently, its consumption is important, as humans are not able to synthesize it or store it at the ocular level [202]. In this sense, the bioavailability and bioavailability of this compound is essential to meet its beneficial effects on health [202]. However, in the case of the ZEA, temperature plays a fundamental role, since thermal processing promotes ZEA release and solubilization in the gastric environment [67]. In addition, its consumption associated with diets or foods rich in fat favors the formation of micelles. These micelles will increase the absorption of the compound at the intestinal level [203]. This is the reason why foods such as sea buckthorn, with a carotenoid-rich oil, possess high bioavailability of ZEA [160]. Thanks to this property, it is relatively easy to increase the bioaccesibility of ZEA, as shown by various studies. One of them endorses the use of coconut oil to increase 6% of ZEA bioaccesibility in goji berries [204]. However, despite the increase in the solubility of ZEA in lipid emulsions, it is necessary to subject the walls of the matrix to microstructural modifications, especially with microalgae, since they can influence the digestibility and bioaccesibility of CA [161]. Nevertheless, microalgae are useful as a source of ZEA in food formulations due to its good bioaccesibility and storage in studies carried out with mice [205]. Additionally, the relationship between ZEA content and bioavailability is another aspect to consider. For example, the bioaccesibility of ZEA in egg yolk is high [206], although the ZEA content is low.

### 3.3. Experimental Studies

The effects of CA on health have been long studied. As mentioned, some CA such as β-cryptoxanthin or β-carotene are precursors of retinol (vitamin A), while others such as fucoxanthin, lutein, or lycopene are not. As such, their intake relates to their role in retinol production, and to their antioxidant, anti-inflammatory, and anti-tumor activities [207]. In this regard, several in vitro as well as in vivo and observational or epidemiologic studies have been carried out in the last decades. Furthermore, the antioxidant role of CA has been long-known and evidenced for its use as antioxidant additive as well as antioxidant test assay [208]. The great majority of studies have assessed the intake of CA to test their effects, as it is the major ingress pathway of these molecules. As with other antioxidants of natural origin with observed health-promoting properties, it has been suggested that the potential chemopreventive effects of these molecules are derived from the synergy of their antioxidant and anti-inflammatory properties, besides their direct inhibition of certain factors involved in cell cycle and apoptosis [209]. This is due to the intimate relationship of oxidative stress as both a cause and result of inflammation and their relationship toward developing cancer [210,211]. Hence, the properties and effectiveness of CA have been tested and evaluated through various ways, both with molecular methods and relating their intake or serum levels with disease or mortality incidence. A summary of relevant findings will be addressed. Experimental designs and outcomes are shown in Table 3.

#### 3.3.1. Observation In Vitro

In vitro experiments testing properties of CA are of great value to analyze the role of specific molecules and discern potential participating molecules. Their apparent results have been reinforced in multiple animals and human studies, while in some cases, results have been mixed. In fact, most experiments with CA have been made in vitro. The in vitro studies analyzed in this article can be divided into two large groups. The first corresponds to those methods that quantify the antioxidant properties of xanthophylls. The second group includes those anti-inflammatory or anti-cancer tests in cell cultures. Inflammatory models usually comprise the use of human or murine macrophage cell cultures and measure differences in the expression or translation of pro-inflammatory mediators such as cytokines (tumor necrosis factor alpha (TNF-α), interleukins (IL)-1β and IL-6), nuclear factor (NF)-κβ (which mediates the expression of these cytokines), and the production of nitric oxide (NO) or enzymes related to the inflammatory process (cyclooxygenase (COX)-2, nitric oxide synthase (iNOS)) [209]. A study on RAW 264.7 murine macrophages, splenocytes, and bone marrow-derived mice macrophages obtained from mice fed with AS reported a significant reduction of IL-1β and IL-6 and generated ROS. Moreover, the authors described that AS inhibit nuclear translocation of NF-κβ and increase the expression of nuclear factor E2-related factor (NRF)-2, which subsequently involves a lower production of reactive oxygen species (ROS) and inflammatory response [217]. Experiments involving FU or some of its metabolites such as fucoxanthinol or apo-9′-fucoxanthinone in vitro have proven anti-inflammatory activities. On murine macrophages RAW 264.7 with a lipopolysaccharide (LPS)-induced inflammation model, FU and fucoxanthin isomers such as 9′-cis or 13′-fucoxanthin all displayed a significant dose-dependent inhibition of pro-inflammatory mediators IL6-IL-1, NO, and TNF-α [212]. Likewise, apo-9′-fucoxanthinone notably reduced levels of NO, ROS, TNF-α, and COX enzyme both in RAW 264.7 macrophages and zebrafish juveniles [213]. A study with different human colon and prostate cancer cell lines elucidated that besides the anti-inflammatory and antioxidant effect of β-carotene, it exerts a direct pro-apoptotic activity on cancerous cells by reducing the expression of caveolin-1 and inducing the activity of several caspases. This protein is heavily involved in cell cycle regulation, and its expression leads to increased protein kinase B levels, being both liable of cell proliferation. Conversely, caspases are signals for apoptosis. The authors were able to elucidate this significant pathway of cell growth inhibition, as this was observed in human colon and prostate cell lines that expressed caveolin-1 (HCT-116, PC-3), but not in those that do not produce it (Caco-2, LNCaP) [229].

#### 3.3.2. Observation In Vivo

Although most of the articles studied dealt with in vitro studies, it is also possible to find various articles about in vivo studies of the activities of xanthophylls. Most of these in vivo studies have been carried out with model animals, including mice, rats, and ferrets. Regarding the results obtained, numerous studies reported that in both animals and humans, retinol levels decrease related to inflammatory responses [230]. For instance, β-cryptoxanthin displayed lower levels of TNF-α, as well as pro-inflammatory transcription factors such as NF-κβ and activator protein (AP)-1. Similarly, another study on the anticancer effect of β-cryptoxanthin on nicotin-induced lung carcinogenesis in mice reported significantly lower levels of IL-6 and AKT alongside the re-expression of tumor-suppressor genes that were silenced by nicotine administration [227]. This interaction between nicotine and β-cryptoxanthin was also analyzed in another in vivo study carried out in this case with ferrets. These ferrets were exposed to cigarette smoke for 3 months in order to induce pulmonary tissue inflammation and carcinogenesis, showing a dose-dependent reduction of both in the groups treated with β-cryptoxanthin [228]. On non-provitamin A CA, dextran sulfate sodium-induced colitis and colon carcinogenesis mice were treated with AS food supplementation. Tissue and gut mucose analysis displayed showed significantly lower NF-κβ, TNF-α, IL-1β, IL-6, iNOS, and COX-2 expression, relating these differences to the near nullification of the induced colitis and a lowered risk of colon carcinogenesis [220]. Regarding FU, which is one of the most promising xanthophylls, a study analyzed the anti-inflammatory activity of injected FU by inducing inflammation with LPS in mice and measuring pro-inflammatory mediators in their aqueous humor. FU exerted a significant reduction of prostaglandin (PG)E-2, NO, and TNF-α levels, also showing a lower infiltration of cells and proteins by the induced inflammation. The most relevant outcome of this study is that the effectiveness shown by FU was highly similar to prednisolone, which was used to establish a feasible comparison [214]. It is noteworthy that most carotenoids display anti-inflammatory and anticancer activities in a dose-dependent fashion, as in cell culture studies.

#### 3.3.3. Observational and Epidemiological Studies

In the last decades, case-control and observational studies have also been carried out in humans to test the effectiveness of CA to extend life expectancy and other health-promoting effects such as reducing the risk of developing cancers, chronic inflammatory diseases, or cardiovascular diseases. Results on the possible chemopreventive effect of CA, especially of β-carotene, are mixed [231]. Nevertheless, this effectiveness has been reported in other studies. Various studies are available, for example, evaluating the potential health-promoting effects of LU. One of them analyzed the effect of LU supplementation in subjects from the Shanghai region with early symptoms of atherosclerosis. Albeit the study was carried out with a small sample (*n* = 65), it was observed that the levels of IL-6, MCP-1, and LDL-cholesterol were significantly lower [221]. In another study, food supplementation with β-carotene, lycopene, and lutein was provided to preterm infants. Although only C reactive was used as an inflammation marker, treated groups displayed significantly lower levels alongside improved retinal development in comparison with the control group [222]. The Alpha-Tocopherol, Beta-Carotene (ATBC) Cancer Prevention Study, which was carried out in 1994 with more than 25,000 (*n* = 29,133) median age male smokers, determined that intake of β-carotene and α-tocopherol supplements could increase the risk of lung cancer, after a ≤8 year follow-up [232]. Additionally, a 24-year follow-up of these subjects did not find a significant chemopreventive effect for supplementing β-carotene toward liver cancer incidence, but it did seem to exert a protective effect in diabetic subjects [233]. However, a recent prospective cohort study of a 30-year follow-up from these subjects determined a significant (*p* < 0.0001) correlation between CA serum levels and reduced all-cause mortality risk in the study quintiles that displayed higher CA in serum as a result of supplement intake, despite their advanced age and smoking habits [234]. These mixed results, also reported in other prospective cohort studies, show a general trend of a protective effect of CA toward cancer development and inflammation, of which research has focused extensively in β-carotene. However, the increased risks of lung cancer development observed in some studies could arguably be due to an excess of retinol in treated groups, as many studies used high-dosage CA supplements as treatment, while subjects may also intake these CA through diet [233]. Taking the case of the ATBC study, the β-carotene dose was of 20 mg, as much as three times the recommended dietary allowance of retinol [232]. Conversely, α-carotene, lycopene, and β-cryptoxanthin have been inversely correlated with developing lung cancer or at least showing a consistent chemopreventive effect [235]. Another study assessed serum CA levels from individuals from the US Third Nutrition and Health Examination Survey (NHANES III) [224], which evaluated health habits and analyzed the serum samples of the participants. In this prospective cohort study, α-carotene and β-cryptoxanthin also displayed effectiveness in lowering the risk of lung cancer development in smokers, but this effect was not apparent in non-smokers [225]. An extensive meta-analysis of human observational studies with a total sample size of more than 150,000 individuals (*n* = 174,067) assessed results from 13 studies, determining that provitamin A CA may exert a protective effect against cancer or cardiovascular mortality [236]. Yet, the authors noted that as mentioned, an excessive production of retinol because of supplementation may be responsible for the reported increased risks of lung cancer development in some case-control studies that considered these variables. It is noteworthy that the greatest meta-analysis up to date to our knowledge evaluated 34 observational studies with a total sample size of 592,479 participants and established correlations between intake or serum levels of α-carotene and lycopene but not β-carotene with lowered risk of developing prostate cancer [237]. These findings also noted that even if these carotenoids had an apparent chemopreventive activity, they were ineffective in preventing malignancy of prostate cancer once it was diagnosed. Altogether, albeit more extensive research with bigger sample sizes and the isolation of potential confusion factors is required, there is a great body of evidence suggesting that in controlled dose ranges, both provitamin A and non-provitamin carotenoids have chemopreventive effects on oxidative stress, inflammation, and cancer development through indirect and direct pathways.

## 4. Algae as Source of Carotenoids

Algae are recognized as a good source for numerous bioactive compounds of great interest, xanthophylls being among them, as reflected on this work. However, the application of these compounds is not linked only to food safety and human health, but factors such as economic costs, efficacy of the designed product, or current legislation are also of vital importance when deciding whether a product it is viable or not and, therefore, it is produced in a commercial way or not. Despite this complexity, algae have become a powerful industry due to its biotechnological applications, advancements in extraction methods, and increasing consumer demand for natural products. As a result, a wide range of products are and have been developed, ranging from nutraceuticals, food additives, or animal feed to drugs or cosmetics [67]. CA play a very important role in all these applications with even better results that their synthetic counterpart [238]. All of these progresses mean that the demand and market of CA are growing significantly, and this year is expected to reach $1.53 billion [239]. Despite this, more advances are still needed to reduce the cost of obtaining it from natural sources. It is estimated that CA derived from algae can reach the cost of $7500/kg [240], whereas synthetic CA could be obtained at roughly half the cost [241]. Nevertheless, despite the great diversity of natural and synthetic CA, only a few of them are commercially produced, including carotenes (β-carotene and lycopene) and xanthophylls (astaxanthin, lutein, zeaxanthin, canthaxanthin, and capsanthin) [242]. Some processes have been developed to increase the benefits. For example, high costs production can be reduced through the development of green technologies as they are considered more profitable, efficient, and ecological, transforming it into an environmentally friendly process [243]. Another important parameter when optimizing is the selection of algae used as source. In this regard, the genomic characterization of these species and identifying relevant target genes involved in CA synthesis and accumulation, paired with efficient culture and harvest techniques; has proven to be an efficient way to maximize CA production [116].

However, there are still barriers that must be solved for the commercialization of CA from algae, such as optimization of their extraction and purification, storage alternatives, and technologies that increase the bioaccessibility and bioavailability of the compounds present in algae [151,157,198]. Currently, different processes such as encapsulation or emulsification arise for CA to achieve their biological functions in humans. In addition, the research has provided data through in vitro and in vivo digestion studies that clarify the absorption mechanism of the different CA, which can be used by industries to improve the formulation of their products. However, more human studies of the nutritional efficiency of these CA extracted from algae are needed [203].

The lack of uniformity of legislation between the different countries makes its study complex. That is why in order to carry out the commercialization of the products obtained, it is necessary to carry out some modifications to adapt them to current legislation. In the case of the EU, as algae were not being used in a traditional or habitual way in food before 15 May 1997, they are considered as novel food as reflected in EU Regulation 2015/2283. This regulation is also applicable to all products obtained from algae such as food supplements of their bioactive components or food additives (*i.e.*, phlorotannins from *Ecklonia cava*) [244]. Therefore, its commercialization request authorization for its incorporation into the market from the European Food Safety Authority (EFSA), which requires health risk studies. These food safety analyses must also be in accordance with current legislation on food safety and food hygiene, respectively included in Regulation (EU) 178/2002 [244] and Regulation (EU) 852/2004 [245], ensuring consumer safety. Moreover, these products can be sold as nutraceuticals without scientific evidence conducted by the EFSA, which is legislated by Regulation CE No. 1924/2006 [246]. However, this same regulation dictates that the health claims alleged to these same products must be backed by proper and significant scientific evidence, which must be submitted to EFSA.

## 5. Conclusions

The use of algae as raw material for obtaining carotenoids, and especially xanthophylls, is an alternative that is gaining interest due to its potential and the bioactivities of the extracted compounds. Currently, CA are used commercially as food additives, feed and nutrient supplements, pigments, and, more recently, as nutraceuticals for cosmetic and pharmaceutical purposes. Despite this, there is little information on the impact of some of these xanthophylls on human health, with most of the studies focusing on FU and AS, which are compounds that also represent the main marine CA. These molecules are characterized by having a high antioxidant activity, and this may be one of the main mechanisms in their anticancer and anti-inflammatory activity. These activities will vary between the different compounds due to the nature of their terminal groups or the length of the chain, among others. However, for these proposals to be viable, it is necessary to carry out a series of advances. These advancements include increased biomass production, increased extraction, and purification performance, as well as reduced implementation costs. Some ways to solve these problems go through genetic engineering or the development of green extraction techniques.

## Figures and Tables

**Table 1:** Xanthophylls in algae: mass production, concentration, and application.

Mol.	Algae	Extraction	Concentration	Applications	Ref.
**FU**	*Fucus vesiculosus*	Enzyme-assisted extraction	0.66 mg/g DW	Development of value-added nutraceutical products from seaweed	[42]
	*Fucus serratus*	Supercritical fluid extraction	2.18 mg/g DW	Obtaining high-purity fucoxanthin	[43]
	*Laminaria japonica*	Microwave-assisted extraction	0.04 mg/g DW	Obtaining high-purity fucoxanthin	[44]
	*Laminaria japonica*	Maceration	0.10 mg/g DW	Drug against chronic kidney disease	[45]
	*Undaria pinnatifida*	Microwave-assisted extraction	0.90 mg/g DW	Obtention of high-purity fucoxanthin	[44]
	*Undaria pinnatifida*	Maceration	3.09 mg/g DW	Scones	[46]
	*Undaria pinnatifida*	Supercritical fluid extraction	0.99 mg/g DW	Carotenoid isolation	[3]
	*Undaria pinnatifida*	Maceration	2.67 mg/g DW	Drug development	[47]
	*Padina tetrastromatica*	Ultrasonic-assisted extraction	0.75 mg/g DW	Nutraceuticals and biomedical applications	[48]
	*Cystoseira hakodatensis*	Maceration	3.47 mg/g DW	Optimization of the environmental conditions	[49]
	*Himanthalia elongata*	Maceration	18.60 mg/g DW	Commercial fucoxanthin production	[50]
	*Tisochrysis lutea*	Ultrasonic-assisted extraction	0.25 mg/g DW	Nutraceutical, cosmetic and pharmaceutical applications, such as for the treatment of metastatic melanoma	[51]
	*Pavlova lutheri*	Ultrasonic-assisted extraction	0.03 mg/g DW	Yogurt	[52]
	*Phaeodactylum tricornutum*	Maceration	0.1 mg/g DW	Milk	[53]
**AS**	*Haematococcus pluvialis*	Conventional extraction	900 kg/2 ha/year	Antioxidant, anti-tumor, anti-inflammatory, ocular protective effect, antidiabetic, coloring agent	[54]
	*Haematococcus pluvialis*	Two-stage system	3.8% dw		[55]
	*Haematococcus pluvialis*	Enzyme	3.6% dw		[56]
	*Haematococcus pluvialis*	Conventional extraction	2–3% dw		[57]
	*Haematococcus pluvialis*	Pressurized extraction	99% of total AS		[58]
**LU**	*Chlorella protothecoides*	Maceration	83.8 mg/L	Antioxidant, light-filtering, eye protection, colorant, potential therapeutic use against several chronic diseases, lower risk of cancer, anti-inflammatory benefits	[59]
	*Chlorella protothecoides*	Mechanical	83.8 mg/L		[60]
	*Chlorella protothecoideswas*	Mechanical	4.92 mg/g		[61]
	*Chlorella vulgaris*	Heptane–ethanol–water extraction	30 mg/g		[62]
	*Scenedesmus almeriensis*	-	0.54% wt		[63]
	*Dunaliella salina*	Conventional extraction	15.4 mg m^−2^ d^−1^		[64]
**ZEA**	*Nannochloropsis oculata*	Supercritical fluids extraction	13.17 mg/g	Antioxidant, anti-inflammatory, eyes and UV light protection, prevention of coronary syndromes, anti-tumoral, anti-cardiovascular diseases, and structural actions in neural tissue	[65]
	*Chlorella ellipsoidea*	Pressurized liquid extraction	4.26 mg/g		[66]
	*Synechocystis sp*	Pulse electric field	1.64 mg/g		[1]
	*Himanthalia elongata*	Pulse electric field	0.13 mg/g		[1]
	*Heterochlorella luteoviridis*	Moderate electric field	244 µg/g		[9]
**CRY**	*Spirulina platensis*	Supercritical fluid extraction	7.5 mg/100 g	Antioxidant, anti-inflammatory, anticancer (lung, oral, pharyngeal), improves respiratory function, stimulation of bone formation and protection, modulation response to phytosterols in post-menopausal women, decreases risk of degenerative diseases	[34,67]
	*Palisada perforata*	Conventional extraction	14.2% total carotenoids		[68]
	*Gracilaria gracilis*	Conventional extraction	10.2% total carotenoids		[68]
	*Pandorina morum*	Maceration	2.38 µg/g DW		[69]
	*Nanochlorum eucaryotum*	Enzyme extraction	-		[70]
**SIP**	*Codium fragile*	Maceration	16 mg/kg fresh algae	Anti-angiogenic, antioxidant, cancer-preventing action; inhibit adipogenesis	[71]
	*Caulerpa lentillifera*	Maceration	0.1% DW		[72]
	*Umbraulva japonica*	Maceration	0.1% DW		[35]
**DIAD**	*Phaeodactylum tricornutum*	MeOH extraction	19% of total pigments	Antioxidant	[73]
	*Phaeodactylum tricornutum*	MeOH extraction	-		[74]
	*Odontella aurita*	EtOH extraction	10% total carotenoids		[75]
	*Phaeodactylum tricornutum*	Whole	14 µg/L		[76]
**DIAT**	*Phaeodactylum tricornutum*	MeOH extraction	17% of total pigments	Antioxidant	[73]

Mol: Molecules/compounds; FU: Fucoxanthin; AS: Astaxanthin; LU: Lutein; ZEA: Zeaxanthin; CRY: β-cryptoxanthin; SIP: Siphonaxanthin; DIAD: Diadinoxanthin; DIAT: Diatoxanthin. dw: Dry weight.

**Table 2:** Delivery systems used to increase marine carotenoids’ bioavailability.

Mol.	Delivery System	Assay	Benefits	Results	Use	Ref.
**FU**	Palm stearin solid lipid core	In vitro	Increase stability during storage	Release of FU of 22.92% during 2 h in SGF and 56.55% during 6 h SIF	Oral supplements	[149]
	Nanoparticles of zein	ABTS DPPH	Increase antioxidant activity	More antioxidant than free FU	Foods and beverages	[150]
	Nanoemulsion	In vitro	Increase stability during storage; antiobesity	95% of FU remains in the emulsion after 4 weeks	Food, beverages, nutraceutics	[151]
	Nanoemulsion (LCT)	In vitro digestion and bioability assays in rats	Increase stability	Increase FU level in serum blood (LCT > MCT)	Functional foods and nutraceutics	[152]
	Chitosan–glycolipid nanogels	In vitro	Significant increase in bioavailability	Lpx levels (nmol MDA/mL) higher in control (30.9) than in emulsions (17.0–12.15)	Foods and nutraceutics	[153]
**AS**	Fish oil	In vitro	Useful for supplementation	Better antioxidant effect	Oral supplements	[154]
	Encapsulation	TBARS Peroxide enzymes	Increase stability	Better antioxidant effect	Foods	[155]
	Pectin–chitosan multilayer	Stability Assays	Increase stability	Better stability than monolayer	Nutraceuticals, functional, medical foods	[156]
	l-lacic acid	Release and stability test	Increase stability	Enhance stability	Functional foods and nutraceutics	[157]
	Ascobyl palmitate emulsion	Stability assay	Sublingual delivery	Enhance sports performance, skin protection, cardioprotective	Dietetic supplementation in sports	[158]
**LU**	β-CD	In vitro	Increase stability	More stable against oxidating agents	Foods	[159]
	Glycyrrhizic acid, arabinogalactan	In vitro	Solubility enhancement	Prevention of H-aggregates formation, increase of photostability	Foods	[159]
**ZEA**	Sea Buckthorn oil and water emulsion	Stability and digestive assays	Increase bioaccesibility	Increase 64.55%	Functional foods and nutraceutics	[160]
	High-pressure treatment	Stability and digestive assays		Improve *Nannochloropsis* sp. ZEA disponibility	Foods	[161]
	Glycyrrhizic acid, arabinogalactan	In vitro	Solubility enhancement	Prevention of H-aggregates formation, increase of photostability	Foods	[159]

SGF: Simulated gastric fluid; SIF: Simulated intestinal fluid; LCT: Long-chain triglycerides; MCT: Medium-chain triglycerides.

**Table 3:** Summary of studies and meta-analysis on the health-related properties and effects of carotenoids and observed results.

Study	Model	Dose	Experimental Design	Observations	Ref.
**Fucoxanthin**					
Anti-inflammatory	In vitro. RAW 264.7 macrophages with LPS-induced inflammation	15–60 μM	Expression of inflammatory mediators	D-d reduction of expression of IL6-IL-1, NO, and TNF-α	[212]
	In vitro (Apo-9′). RAW 264.7 macrophages and zebrafish model	25–100 μg/mL	Reduction of LPS-induced inflammation	D-d reduction of NO, ROS, TNF-α, and COX production	[213]
	In vitro and in vivo. RAW 264.7 and aqueous humor of rats	10 mg/kg	Reduction of LPS-induced inflammation	D-d reduction of PGE2, NO, TNF-α by inhibiting iNOS and COX-2	[214]
Anti-cancer	Ex vivo. B16F10 cell culture implanted in mice	200 μM	Growth inhibition of melanoma	D-d growth inhibition by inducing G_0_/G_1_ cell cycle arrest and apoptosis; inhibition production of retinoblastoma protein	[215]
	In vitro. Human leukemic HL-60 cells	15.2 μM	Inhibited the proliferation	DNA fragmentation	[216]
**Astaxanthin**					
Anti-inflammatory	In vitro. RAW 264.7, splenocytes, and bone-narrow macrophages	25 μM	Expression of inflammatory mediators in LPS-induced inflammation	D-d significant reduction of IL-6, IL-1β, and ROS production	[217]
	In vivo. Mice with induced acute lung injury	60 mg/kg/day for 14 days	Analysis of inflammation markers, tissue damage	Significant reduction of mortality, histological damage, inflammatory infiltration, and iNOS and NF-κβ levels	[218]
Anti-cancer	In vitro. Human colon cancer lines HCT-116, SW480, WiDr, HT-29 and LS-174	5–25 µg/mL	Growth inhibition of with *H. pluvialis* astaxanthin-rich extract	D-d cell cycle arrest and apoptosis induction by lowering expression of Bcl-2, AKT and induced expression of apoptotic MAPK	[219]
	In vivo. Chemically induced colitis and colon carcinogenesis mice	200 ppm	Analysis of inflammatory biomarkers	D-d inhibition of NF-κβ, TNF-α, IL-1β, IL-6, and COX-2 expression; lower iNOS expression at high dosage	[220]
**Lutein**					
Anti-inflammatory	Observational study. Early atherosclerosis patients (*n* = 65)	20 mg/day for 3 months	Differences in serum cytokines, and metabolic biomarkers	Significant reduction in serum IL-6 MCP-1 and LDL-cholesterol after 3 months of supplementation	[221]
	Observational study. Preterm infants (*n* = 203)	30 mL/ kg/ day until 40 weeks post-menstrual age	Differences in inflammation biomarkers	Enhanced retinal development and reduced C-reactive protein levels	[222]
Anti-cancer	In vivo. Rats	3–30 g/L	Inhibition of N-methylnitrosourea-induced colon crypt foci formation	Significantly lowered formation of aberrant crypt foci	[223]
**β-cryptoxanthin**					
Anti-cancer	Prospective cohort study. Smokers and non-smokers from NHANES III (*n* = 10,382)	Dietary contribution	20-year cohort	Higher serum levels of β-CRY were associated with lower death risk, but not for non-smokers	[224,225]
	Ex vivo. Human gastric cell lines AGS and SGC-7901 implanted in mice	0–40μM	Growth and proliferation inhibition	D-d growth and proliferation inhibitory activity by reducing cyclins, endothelial growth factor, PKA and increasing cleaved caspases expression	[226]
	In vivo. Mice	10 mg/kg diet	Induced emphysema and lung tumorigenesis	D-d tumor mass reduction, decreased levels of IL-6 and AKT and restoration of silenced tumor-suppressor genes	[227]
	In vivo. Cigarette smoke-exposed ferrets	7.5–37.5 μg/kg/day	Inflammation biomarkers and tissue damage analysis	D-d inhibition of NF-κβ, TNF-α, AP-1 expression as well as lung tissue squamous metaplasia and inflammation	[228]
**Siphonaxanthin**					
Anti-cancer	In vitro. Human leukemia (HL-60) cells	5–20 μM	Analysis on cell viability and apoptosis	D-d reduction of cell viability and induction of apoptosis by increasing levels of DR5, lower expression of Bcl-2 and increase in caspase-3	[129]

D-d: Dose-dependent; LPS: Lipoplysaccharide, ROS: Reactive oxygen species, IL: Interleukin, NRF2: Nuclear factor E2-related factor 2, PKA: Protein kinase A, AKT: Protein kinase B, ERK: Extracellular signal-regulated kinase, PAI-1: Plasminogen activator inhibitor-1, MMP: Metalloproteinases, Bcl-2: B-cell lymphoma 2, PG: Prostaglandin, RR: Relative risk, CI: Confidence interval.

## Data Availability

Not applicable.
